# Prevalence of Metabolic‐Associated Steatotic Liver Disease in Patients With Primary Aldosteronism

**DOI:** 10.1111/cen.15231

**Published:** 2025-03-13

**Authors:** Irene Tizianel, Alberto Madinelli, Filippo Crimì, Mattia Barbot, Simona Censi, Chiara Sabbadin, Filippo Ceccato

**Affiliations:** ^1^ Department of Medicine DIMED Endocrine Unit Padua Italy; ^2^ Endocrine Unit, University‐Hospital of Padova Padua Italy; ^3^ Institute of Radiology, University‐Hospital of Padova Padua Italy

**Keywords:** adrenal incidentaloma, computed tomography, metabolic associated steatotic liver disease, mild autonomous cortisol secretion, primary aldosteronism

## Abstract

**Objective:**

To assess the prevalence of metabolic associated steatotic liver disease (MASLD) in patients with primary aldosteronism (PA) compared to benign adrenal adenomas, and to evaluate the impact of hormonal excess in inducing MASLD.

**Design:**

Single‐centre retrospective study.

**Methods:**

Hepatic steatosis was assessed by liver/spleen (L/S) ratio from unenhanced abdomen computed tomography images (reference value < 1.1) in a cohort of 41 patients with PA without cortisol cosecretion, 20 unilateral (uPA) and 21 bilateral (BPA), 50 with nonfunctioning adrenal incidentalomas (NF‐AI), 48 with mild autonomous cortisol secretion (MACS) and 10 with adrenal Cushing Syndrome (CS).

**Results:**

Hepatic steatosis was increased in patients with PA at diagnosis: L/S ratio was lower in PA than NF‐AI (1.1 vs. 1.25, *p* < 0.001) and MACS (1.1 vs. 1.21, p 0.007), but was similar to adrenal CS (1.1 vs. 1.15, *p* = 0.147). A improvement in L/S ratio after medical or surgical treatment was observed in PA patients, resulting in reduced liver steatosis. MASLD prevalence was higher in PA compared to MACS (49% vs. 25%, *p* < 0.05) and NF‐AI (49% vs. 14%, *p* < 0.001), but similar to CS (49% vs. 45%, *p* = 0.61). uPA patients had higher MASLD prevalence compared to BPA group 71% (53%–89%) versus 25% (7%–43%).

**Conclusions:**

Prevalence of MASLD was increased in PA (higher in uPA than BPA) compared to MACS and NFAI, and similar to adrenal CS.

## 1 Introduction

Primary aldosteronism (PA) is a group of disorders characterised by inappropriately high aldosterone secretion for sodium levels and volemic status, autonomous from renin‐angiotensin system regulation and serum potassium concentration. PA is the most common endocrine form of secondary hypertension, with an estimated prevalence of 10% in hypertensive patients and even higher (20%–30%) among those with resistant hypertension. It is frequently caused by unilateral adrenal adenoma (uPA) or bilateral (BPA) adrenal hyperplasia [1].

This impaired mineralocorticoid activity in the setting of PA has multiple systemic implications, which lead to increased cardiovascular morbidity: arterial hypertension, left‐ventricular hypertrophy, myocardial fibrosis, atrial fibrillation, and impaired glucose metabolism [1, 2, 3]. Despite the well‐known role of aldosterone excess in cardiovascular disease, novel data suggest that mineralocorticoid excess drives metabolism towards diabetes mellitus (DM): PA patients have a higher prevalence of DM compared to both normotensive and hypertensive subjects [4]. An abnormal glucose metabolism due to insulin resistance seems to be linked to aldosterone excess, which acts in several insulin‐target organs, such as the liver, skeletal muscle, and adipose tissue. Other concurrent environmental factors, such as hypokalemia, may impair insulin action [5]. After adrenalectomy, patients with uPA display a regression of glucometabolic complications, with a significant reduction in both plasma glucose and insulin levels during oral glucose load [6]. The prevalence of nonalcoholic steatotic liver disease (NAFLD) is higher in PA patients, with hypokalemia as a principal predictor of its development. However, the underlying pathogenetic mechanism has not been elucidated yet [7, 8]. Mineralocorticoid receptor (MR) activation in cardiovascular tissues leads to hypertension, inflammation, and fibrosis, overlapping those mechanisms involved in the development of liver disease [9, 10].

Recently, a new definition for steatotic liver disease, called metabolic dysfunction‐associated steatotic liver disease (MASLD), has been proposed by panels of international experts, as a more appropriate definition of steatotic liver disease, associated with metabolic dysfunction [11, 12]. MASLD is diagnosed with the presence of hepatic steatosis ≥ 5% (detected by either imaging, blood biomarkers, or liver histology) with at least one of overweight/obesity (BMI ≥ 25 kg/m^2^) or type 2 DM. In case of normal weight (BMI < 25 kg/m^2^) and absence of type 2 DM, at least two metabolic dysfunctions must be present: (1) waist circumference > 90 cm or 80 cm in men or women, respectively; (2) arterial blood pressure ≥ 130/85 mmHg or specific drug treatment; (3) plasma triglycerides > 150 mg/dL or specific drug treatment; (4) plasma HDL cholesterol < 40 mg/dl in men or < 50 mg/dl in women or specific drug treatment; (5) prediabetes (fasting plasma glucose levels 100–125 mg/dL) or 2‐h post load glucose levels 140–199 mg/dL or HbA1c 5.7%–6.4%; (6) homoeostasis model assessment of insulin resistance > 2.5; (7) plasma high‐sensitivity C‐reactive protein level > 2 mg/L [13].

The most relevant innovation that distinguishes MASLD from NAFLD is that concomitant liver diseases (especially alcohol intake) is no longer required as exclusion criteria to entertain the diagnosis, more than the demonstration of metabolic dysregulation. MASLD screening should be considered in patients with PA because multiple comorbidities (arterial hypertension, left ventricular hypertrophy, endothelial dysfunction, and metabolic alterations) increase their cardiovascular risk [14, 15].

The aims of the study were: (a) to assess the prevalence of MASLD in PA patients compared to adrenal adenomas with various degrees of cortisol excess (MACS and CS) and NF‐AI; (b) to evaluate the impact of hormonal excess in inducing MASLD.

## 2 Materials and Methods

### 2.1 Study Design and Patients

We conducted a single‐centre study at Padova University Hospital, approved by the local Ethics Committee (study PITACORA, Pituitary Thyroid Adrenal tumours cardiovascular and outcome related long‐term Assessment, protocol number AOP3318, Ethic Committee registration 5938‐AO‐24). All data are available in the Repository of the University of Padova. [16] This observational study was conducted in accordance with the STrengthening the Reporting of OBservational studies in Epidemiology (STROBE) guidelines [17] (Figure S1).

All patients had a confirmed PA diagnosis and subtype differentiation by adrenal venous sampling (AVS), according to the Endocrine Society Guidelines [1]. PA was diagnosed with the following conditions: baseline plasma aldosterone concentration (PAC) ≥ 415 pmol/L (15 ng/dL), aldosterone to renin ratio (ARR) ≥ 91 pmol/L/mU/L, and PAC after saline infusion test ≥ 277 pmol/L (10 ng/dL) and/or PAC suppression after captopril challenge test < 30%. Clinical and biochemical outcomes were evaluated according to the primary aldosteronism surgical outcome criteria (PASO) [18].

Liver steatosis was assessed on computed tomography (CT) scans performed at PA diagnosis, mineralocorticoid receptor antagonist (MRA) treatment before the diagnosis of PA was considered an exclusion criterion. All PA patients presented with arterial hypertension and were studied for cortisol cosecretion: to focus on aldosterone secretion, adequate cortisol suppression after 1 mg dexamethasone (DST, cortisol < 50 nmol/L) was an inclusion criterion. Subsequent PA treatment (unilateral adrenalectomy or MRA therapy) was guided by AVS results and according to patients' preferences. From our cohort of 79 patients with PA, 41 fulfilled the inclusion criteria: 21 were defined unilateral PA (uPA), and 20 were classified as bilateral PA (BPA), according to AVS results.

As controls, we randomly selected 50 hypertensive patients with nonfunctioning adrenal incidentalomas (NF‐AI), 48 with mild adrenal autonomous cortisol secretion (MACS), and 10 with overt Cushing Syndrome (CS) in our cohort of 568 patients with benign adrenal adenoma.

All adrenal incidentalomas were characterised by high lipid content with Hounsfield Unit (HU < 10), at least 36 months of follow‐up, normal aldosterone to renin ratio (ARR), and urinary fractionated metanephrines. NF‐AI were characterised by suppressed serum cortisol (≤ 50 nmol/L) after 1‐mg DST (adequate dexamethasone levels were assessed in all tests) and normal ACTH levels (> 10 ng/L) at baseline and last follow‐up visit. The 48 patients with MACS were defined by unsuppressed cortisol after 1 mg DST (> 50 nmol/L), low/suppressed ACTH levels, urinary free cortisol (UFC) and late‐night salivary cortisol (LNSC) below the upper limit of normality (ULN). Patients with adrenal CS had clinical manifestations related to cortisol excess and biochemical diagnosis of ACTH‐independent CS: cortisol after 1 mg DST (> 50 nmol/L), suppressed ACTH levels (< 10 ng/L), UFC > 2 ULN and loss of circadian cortisol rhythm (LNSC > ULN), histological confirmation of adrenal adenoma and hypercortisolism remission after surgery [19].

### 2.2 Liver Steatosis and MASLD Assessment

Liver and spleen CT attenuation, measured in HU, was obtained from unenhanced abdomen CT images available in the Picture Archiving and Communication System (PACS). All the CT examinations were performed with a 64‐slice CT scanner (Somatom Sensation, Siemens Healthineers, Erlangen, Germany) with the following parameters: craniocaudal image acquisition with tube voltage of 120 kV, 250 mAs effective dose, rotation time of 0.5 s, 0.6 mm detector collimation, and 0.75 pitch. The slice thickness for unenhanced scans was 1.5 mm, and the reconstruction kernel was 30B. The liver was divided into five sections, while the spleen was divided into three sections. In each one, a standard 1.0 cm^2^ region of interest (ROI) was selected. During ROI selection, homogeneous areas representative of the parenchyma were visually selected, avoiding vessels, margins, lesions, and bile ducts [20]. The measurements were performed by two experienced radiologists, in consensus.

We adopted the reference value of 1.1 for the liver/spleen (L/S) ratio to detect at least moderate steatosis, thus involving more than 30% of the liver parenchyma, because it has been shown to provide the best diagnostic accuracy [21, 22, 23].

**Figure 1:**
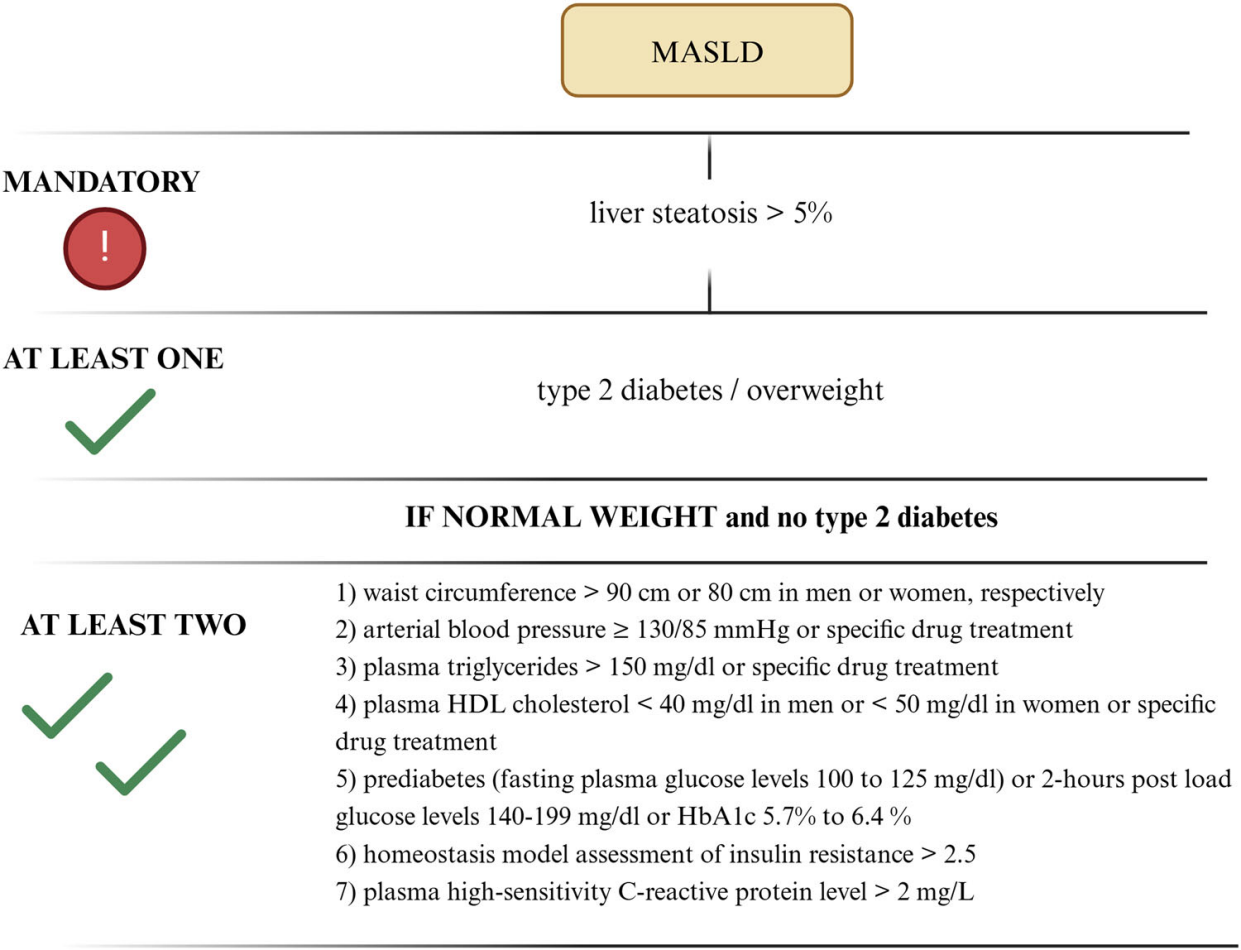
MASLD definition criteria. Created with biorender.com.

We assessed the prevalence of MASLD in PA, MACS and adrenal CS defined as: hepatic steatosis (L/S ratio ≤ 1.1) with at least one of overweight/obesity (BMI ≥ 25 kg/m^2^) or type 2 DM or, in case of normal weight (BMI < 25 kg/m^2^) and absence of type 2 diabetes, the detection of at least two additional metabolic dysfunctions (Figure 1) [11].

### 2.3 Statistical Analysis

The normality of distribution for each variable was assessed using the Kolmogorov‐Smirnov test. Continuous variables were summarised using mean and standard error (SE) for normally distributed data or median and interquartile range (IQR) for non‐normally distributed data. Categorical variables were presented as counts, frequencies, and proportions within each category. Mann–Whitney U test and Wilcoxon Signed‐Rank test were used to compare two groups or two related samples, respectively. A Kruskall‐Wallis H‐test, followed by a post hoc test for subgroup comparison, was used to compare continuous data in more than two groups.

Categorical variables were compared using the chi‐square test or Fisher's exact test, as appropriate. Multiple comparisons for qualitative variables were made with Bonferroni correction. Regression analysis was performed with binary logistic regression models to assess the association between the final diagnosis as dependent variable, and clinical and biochemical variables as predictors. Adjusted logistic regression models were based on clinical relevance, collinearity and interactions. Statistical significance was defined as a *p*‐value < 0.05. All statistical analyses were conducted using SPSS statistical software (version 24.0, SPSS Inc. Chicago, IL).

## 3 Results

Clinical and biochemical characteristics of PA patients showed that uPA (*n* = 21) and BPA (*n* = 20) groups at diagnosis were similar for age, aldosterone and renin levels, BMI, and glucose metabolism (GM) alterations distribution. Potassium levels at diagnosis were lower in uPA compared to BPA (2.8 vs. 3.6 mmol/L, respectively, *p* < 0.001), as shown in Table 1.

**Table 1:** Clinical and biochemical characteristics in patients with unilateral (uPA) and bilateral (BPA) primary aldosteronism.

	uPA (tot *n* = 21)	BPA (tot *n* = 20)
**Age at diagnosis (median)**	47 (34.5–53.8)	48.5 (34.8–61.8)
**BMI (< 25/25–30/> 30 kg/m** ^**2**^ **)**	6/7/3	5/6/1
**GM (normal/IFG/DM)**	1/15/3	6/10/5
**Aldosterone at diagnosis (median)**	921.5 pmol/L (559.5–1399.0)	818 pmol/L (607.8–1429.8)
**Renin at diagnosis (median and IQR)**	2 miU/L (1.0–2.18)	1 miU/L (1.0–1.80)
**K** ^**+**^ **at diagnosis (median and IQR)**	2.8 mmol/L (2.6–3.0)^a^	3.6 mmol/L (3.3–3.7)
**MASLD (n°/tot, percentage)**	15/21^b^ (71%)	5/20 (25%)

*Note:* Data are presented as medians and interquartile range (in brackets) or absolute numbers and percentages.

Abbreviations: BMI, body mass index; DM, diabetes mellitus; F, female; GM, glucose metabolism; M, male.

^a^ *p* < 0.001.

^b^ *p* < 0.05.

Epidemiological and clinical data of multiple groups comparison (PA, NFAI, MACS and CS) are reported in Table 2. Gender distribution and glucose metabolism abnormalities were similar between the groups. As expected, patients with NF‐AI and MACS were older than PA and CS.

**Table 2:** Compared analysis between groups.

	PA (tot *n* = 41)	NF‐AI (tot *n* = 50)	MACS (tot *n* = 48)	CS (tot *n* = 10)	p value
Age at diagnosis (median)	48 (42–57.5)	65 (54–69.5)^a^	68.5 (64.25–73)^a^	42 (38.75–58.25)^b^^,^ c	< 0.001
Gender F/M (% F)	19/22 (46%)	29/21 (58%)	29/19 (60%)	9/10 (90%)	0.086
GM (normal/IFG/DM)	25/8/7 (62/20/18%)	36/5/9 (72/10/18%)	32/1/15^a^ (67/2/31%)	0/6/4^a^ (0/60/40%)	0.085
BMI (< 25/25–30/> 30 kg/m^2^)	13/13/4 (43/43/13%)	22/28/0^a^ (44/56/0%)	24/24/0^a^ (50/50/0%)	2/7/1 (20/70/10%)	0.015
Liver to spleen (L/S) ratio at diagnosis	1.10^d^ (0.77–1.32)	1.25^e^ (0.91–1.62)	1.21 (0.76–1.60)	1.15 (0.99–1.31)	< 0.001
MASLD (n°/tot, percentage)	20/41 (49%)	7/50 (14%)^f^	12/48 (25%)^a^	4/10 (40%)	0.001

*Note:* Data are presented as median and interquartile range (in brackets) or absolute number and percentage.

Abbreviations: BMI, body mass index; DM, diabetes mellitus; F, female; GM, glucose metabolism; M, male.

^a^ *p* < 0.05 versus PA.

^b^ *p* = 0.05 versus NF‐AI.

^c^ *p* = 0.05 versus MACS.

^d^ *p* < 0.001 versus NF‐AI.

^e^ *p* < 0.01 versus MACS.

^f^ *p* < 0.001 versus PA.

We assessed MASLD prevalence in our study population and compared the different groups: MASLD was more common in patients with uPA compared to BPA (71% vs. 25% *p* < 0.001, Table 1). Conversely, PA was characterised by a higher prevalence of MASLD compared to MACS (PA 49% vs. MACS 25%, *p* < 0.05) and NFAI (14%, *p* < 0.001), but similar to adrenal CS (40%, p 0.487). Table S1 reports the complete metabolic description of the 149 patients of the study [16].

To assess the impact of hormonal excess in MASLD determination in patients with PA we evaluated the prevalence of GM alterations in the different subgroups related to BMI: normal GM/IFG/DM prevalence was not otherwise distributed for BMI (normal‐weight/overweight/obesity, *p* = 0.053). We also assessed median potassium levels between the different groups: PA 3.3 mmol/L (IQR 2.8–3.6), NF‐AI 4 mmol/L (IQR 3.8–4.3) MACS 4 mmol/L (IQR 3.8–4.2) and CS 3.6 mmol/L (IQR 3.5–4.1). Median potassium levels were lower in PA versus CS, NF‐AI and MACS (respectively *p* = 0.014, *p* < 0.001 and *p* < 0.001).

### 3.1 Liver Steatosis by L/S Ratio

The degree of hepatic steatosis was assessed by measuring the L/S ratio on unenhanced baseline CT images, reassumed in Table 2. The L/S ratio was lower in PA versus NF‐AI (median L/S ratio 1.10 vs. 1.25, *p* < 0.001) and versus MACS (median L/S ratio 1.10 vs. 1.21, *p* = 0.007). On the contrary, the L/S ratio at diagnosis in the adrenal CS group was similar to that of PA patients (median L/S adrenal CS 1.15, *p* = 0.147).

A multivariable logistic regression analysis was performed to assess predictors for hepatic steatosis, defined by L/S ratio < 1.1, differentiating PA and CS groups, as shown in Tables 3 and 4. In the subgroup of PA patients, BMI was a predictor of hepatic steatosis. On the contrary, aldosterone levels, glucose metabolism alterations and potassium levels were nonsignificant (Table 3). Considering PA patients after treatment (either medical or surgical) BMI distribution was no more significant in predicting hepatic steatosis (OR 0.001, 95% CI 0.00–0.005 *p* = 0.99) and BMI distribution was similar in PA patients at diagnosis and after treatment (*p* = 0.845). No predictors of hepatic steatosis were found in patients with adrenal CS (Table 4).

**Table 3:** Multivariable logistic regression analysis in PA for prediction of hepatic steatosis at diagnosis (L/S < 1.1).

	OR	95% CI	p
**GMA**	0.49	0.07–3.45	0.48
**BMI** > **25 kg/m** ^**2**^	9.21	1.31–64.63	0.02
**Aldosterone at diagnosis**	0.998	0.996–1.05	0.06
**Hypokalemia**	1.44	0.22–9.52	0.70

Abbreviations: BMI, body mass index; GMA, glucose metabolism alterations; L/S, liver to spleen.

**Table 4:** Multivariable logistic regression analysis in CS for prediction of hepatic steatosis at diagnosis (L/S ratio < 1.1).

	OR	95% CI	p
**GMA**	0.71	0.12–4.21	0.70
**BMI** > **25 kg/m** ^**2**^	1.02	1.09–3.25	0.37
**Cortisol after 1 mg DST**	1.09	0.98–1.21	0.13
**Hypokalaemia**	0.49	0.06–4.02	0.51

Abbreviations: BMI, body mass index; DST, dexamethasone suppression test; GMA, glucose metabolism alterations; L/S, liver to spleen.

### 3.2 L/S Ratio Before and After Treatment in PA Patients

Longitudinal evaluation before and after treatment was available in a subgroup of 21 patients with PA: endocrine data and L/S ratio were collected at diagnosis and at least 6 months after treatment, either unilateral adrenalectomy (in nine patients, PA^surgery^) or MRA therapy (in 12 patients, PA^MRA^).

The median L/S ratio at diagnosis was 1.1 (IQR 1–1.22) and the median L/S ratio posttreatment was 1.26 (IQR 1.13–1.36), indicating a reduction of liver steatosis (Z = −4.017, *p* < 0.001). A subgroup analysis of only PA patients with hepatic steatosis (L/S ratio < 1.1) revealed no difference in the L/S ratio before and after PA treatment, while the improvement of L/S ratio after treatment remained significant after differentiating for surgical or medical treatment (Table 5).

**Table 5:** Liver to spleen (L/S) ratio pre‐ and posttreatment in PA patients treated with surgery (PA^surgery^) or mineralocorticoid receptor antagonist (PA^MRA^).

	L/S ratio pre (median)	L/S ratio post (median)	ΔL/S ratio	L/S ratio post –L/S ratio pre
**PA** ^**surgery**^ **(*n*** = **9)**	1.06 (1–1.22)	1.22 (1.13–1.36)	0.15	Z = −2.71, *p* = 0.018
**PA** ^**MRA**^ **(*n*** = **12)**	0.88 (0.83–1.05)	1.10 (1.02–1.31)	0.14	Z = −2.023, *p* = 0.043

*Note:* Data are presented as medians and interquartile range (in brackets).

## 4 Discussion

PA patients are at high risk of cardiovascular complications secondary to aldosterone excess which is responsible to target organ damage independently of the degree of arterial hypertension [24]. PA is also characterised by GM impairment, primarily due to insulin resistance, and a higher incidence of metabolic syndrome and type 2 DM [4, 5, 25]. The pathogenetic mechanisms of insulin resistance in PA are not entirely clarified yet; however, it seems that aldosterone exerts a role on insulin‐target tissues through genomic and non‐genomic effects via MR and glucocorticoid receptor (GR) [2, 4].

MASLD, a new definition of steatotic liver disease, affects almost a quarter of the world's adult population: it is a global health challenge [11], and new specific drugs have been developed [26]. Despite the well‐known role of obesity and insulin resistance in determining liver steatosis, there are important relationships between MASLD and endocrinopathies [27].

PA has been reported to be associated with a higher prevalence of steatotic liver disease and metabolic syndrome, compared to normotensive subjects or patients with essential hypertension [28, 29, 30]. A close relationship between overt cortisol excess in CS [31], subclinical cortisol excess in MACS [32], and autonomous aldosterone secretion with higher cardiovascular risk, is reported. Furthermore, an increased cardiometabolic risk has been reported also in patients with NF‐AI [33].

In our study, we observed a heightened MASLD prevalence in the cohort of PA (49%), especially in uPA than BPA. It has been reported that aldosterone excess decreases glucose‐stimulated insulin secretion in a dose‐dependent manner, concomitantly with reduced C‐peptide levels, during hyperglycemic clamps in mice and humans [34]. Furthermore, MRA treatment can improve glucose tolerance and lipid metabolism in different mouse models of obesity [35]. Hypokalemia is a main determinant of decreased insulin secretion in PA, through a mechanism of membrane depolarisation and closure of voltage‐gated calcium channels secondary to the opening of ATP‐sensitive potassium channels [2]. Fallo et al. reported ultrasonographic hepatic steatosis as a frequent finding in PA, especially in hypokalemic patients characterised by higher insulin resistance, with similar results between uPA and BPA [8]. On the contrary, a Japanese study in 2020 reported similar liver steatosis (defined by L/S ratio < 1.0) between uPA and BPA (21.2% vs. 19.6%) [31].

Our finding of higher MASLD prevalence in uPA can be related to the lower potassium levels observed, supporting known evidence that hypokalemia is a stronger determinant of MASLD pathogenesis [2, 7]. The crucial role of hypokalemia in steatotic liver disease development is reported by Chen et al. potassium supplementation partially improved inflammatory and metabolic profiles in PA patients. The authors also reported a relatively higher prevalence of steatotic liver disease (44% vs. 27%) and DM (19.8% vs. 9.9%) in hypokalemic compared to normokalemic PA patients [7].

To assess the role of hormonal excess in MASLD determination we compared BMI and GM alterations prevalence in our cohorts. We found similar prevalence of GM alterations between normal‐weight/overweight and obese patients, suggesting that metabolic alterations may not be considered the only determinant in MASLD prevalence: hormonal excess may play a role.

A recent Chinese study reported a higher prevalence of steatotic liver disease in PA (35%) than in non‐PA patients (29%), even if the distribution of obesity, dyslipidemia, and insulin resistance was similar between the two groups. In addition, hypokalemic PA patients had a worse metabolic status than normokalemic ones [7]. The latter finding corroborates the impact of aldosterone excess in metabolic disturbances and MASLD development.

We found out that MASLD prevalence was higher in PA compared to MACS, but similar to that of CS patients. Moreover, CS had higher MASLD prevalence than MACS (40% vs. 25%, respectively) and NF‐AI patients (40% vs. 14%, respectively). This finding seems to highlight the impact of the overt form of hormonal excess, both aldosterone and cortisol, in determining MASLD, compared to subclinical hormonal excess (in MACS). One hypothesis may regard the higher transcriptional activity of hormonal receptors in overt forms of hormonal excess, secondary to the hormone‐receptor binding. It is well known that aldosterone and cortisol excess promote steatotic liver disease through different mechanisms: impaired insulin action, increased lipolysis and gluconeogenesis. In literature, NAFLD prevalence in overt CS was reported around 20%, correlated with total abdominal and visceral fat area [29].

We also compared potassium levels in the different study groups, and we found that PA patients had lower potassium compared to CS patients. Therefore, since similar MASLD prevalence in these two groups, overt cortisol excess in CS seems to have a relevant role in MASLD occurrence, even in the absence of hypokalemia, probably related to a local (hepatic) mineralocorticoid effect of cortisol excess.

Cortisol excess is implicated in the development of hyperglycaemia, insulin resistance, dyslipidemia, and obesity, as well as NAFLD. Moreover, it increases lipolysis and muscle protein catabolism to provide substrates for hepatic glucose production. The enhanced lipolysis results in increased free acids which are directly employed by the liver, with a consequent increase in triglycerides synthesis and hepatic steatosis [36].

In our study, cortisol cosecretion was excluded in all PA patients. Some authors showed that PA patients without cortisol cosecretion still have higher HOMA‐IR and DM prevalence than general population‐matched controls, indicating that aldosterone impairs insulin sensitivity and secretion per se [37, 38]. A recent study by Mansour et al. evaluated the effect of mild cortisol cosecretion on body composition and metabolic parameters in patients with PA. They found no differences in total, visceral, and subcutaneous fat volumes in PA patients with or without concomitant mild cortisol excess [39]. MR is involved in gene regulation of hepatic glucose production [10], and aldosterone induces 11‐β‐hydroxysteroid dehydrogenase type 1 expression upon MR activation, amplifying local GC action [34]. All these aspects can explain our finding of higher MASLD prevalence in PA compared to MACS, given the direct contribution of aldosterone excess through hepatic MR in regulating gluconeogenesis but also activating proto‐oncogene tyrosine protein kinase, which produces reactive oxygen species (ROS) enhancing proteasome‐dependent degradation of insulin receptor substrate [2]. Chronic hypercortisolism increases lipogenesis and gluconeogenesis regulating hepatic GR activity, leading to steatotic liver disease [36, 40].

Therefore, concerning our findings, we can assume that cortisol excess configuring CS exerts a key role, similar to that of aldosterone, in determining MASLD, whereas mild cortisol excess without clinical features of CS (that characterises patients with MACS), has a lower impact on MASLD onset.

Finally, within a subgroup of our PA patients we reported an improvement in L/S ratio at least 6 months after PA treatment, either medical or surgical. No difference in L/S ratio reduction in PA^surgery^ versus PA^MRA^ groups was observed. These findings support the crucial role of aldosterone excess in MASLD development and highlight the potential improvement in hepatic steatosis.

Regarding these topics, a recent study on steatotic liver disease in Cushing syndrome reported a MASLD prevalence of 26.5% in a population of 49 patient with either ACTH‐dependent or independent Cushing syndrome. The authors reported that MASLD was only associated with metabolic factors, irrespective of cortisol secretion. However, as in our PA cohort, the evaluation of steatotic liver disease after CS treatment showed an improvement of liver steatosis [41].

The main limitations of this study are its retrospective design, the small number of patients evaluated before and after treatment in a longitudinal perspective, and the lack of a control group. Therefore, prospective studies are needed to fill these gaps.

## 5 Conclusions

In our study we reported a considerable prevalence of MASLD in PA, higher in patients with uPA compared to BPA, probably reflecting lower potassium levels. We also described a significative difference between PA and NF‐AI/MACS concerning MASLD prevalence, while MASLD prevalence in PA and adrenal CS was comparable, and the improvement of liver steatosis after treatment.

## Author Contributions

I.T. and A.M.: data collection; F.C. and I.T.: data analysis; F.C., C.S. and I.T.: writing of original draft; S.C. and M.B.: supervision, writing‐review and editing. All authors approved the final version of the paper.

## Consent

Informed consent was obtained in all patients.

## Conflicts of Interest

The authors declare no conflicts of interest.

## Supporting information

Supporting information.

## Data Availability

All data generated or analysed during this study are included in this published article, or in the repository data https://researchdata.cab.unipd.it/id/eprint/1490, DOI: 10.25430/researchdata.cab.unipd.it.00001274.
