# Supplementary material for: Prevalence of Metabolic‐Associated Steatotic Liver Disease in Patients With Primary Aldosteronism
Source: Clin Endocrinol (Oxf). 2025 Mar 13;102(6):618–25. doi: 10.1111/cen.15231 (PMC12046541; doi:10.1111/cen.15231)
Supplement: Supplementary file 1 — Supporting information. [file CEN-102-618-s001.pdf]

Prevalence of metabolic-associated steatotic liver disease in patients with primary aldosteronism

Supplementary Figure 1. STROBE flowchart of the study

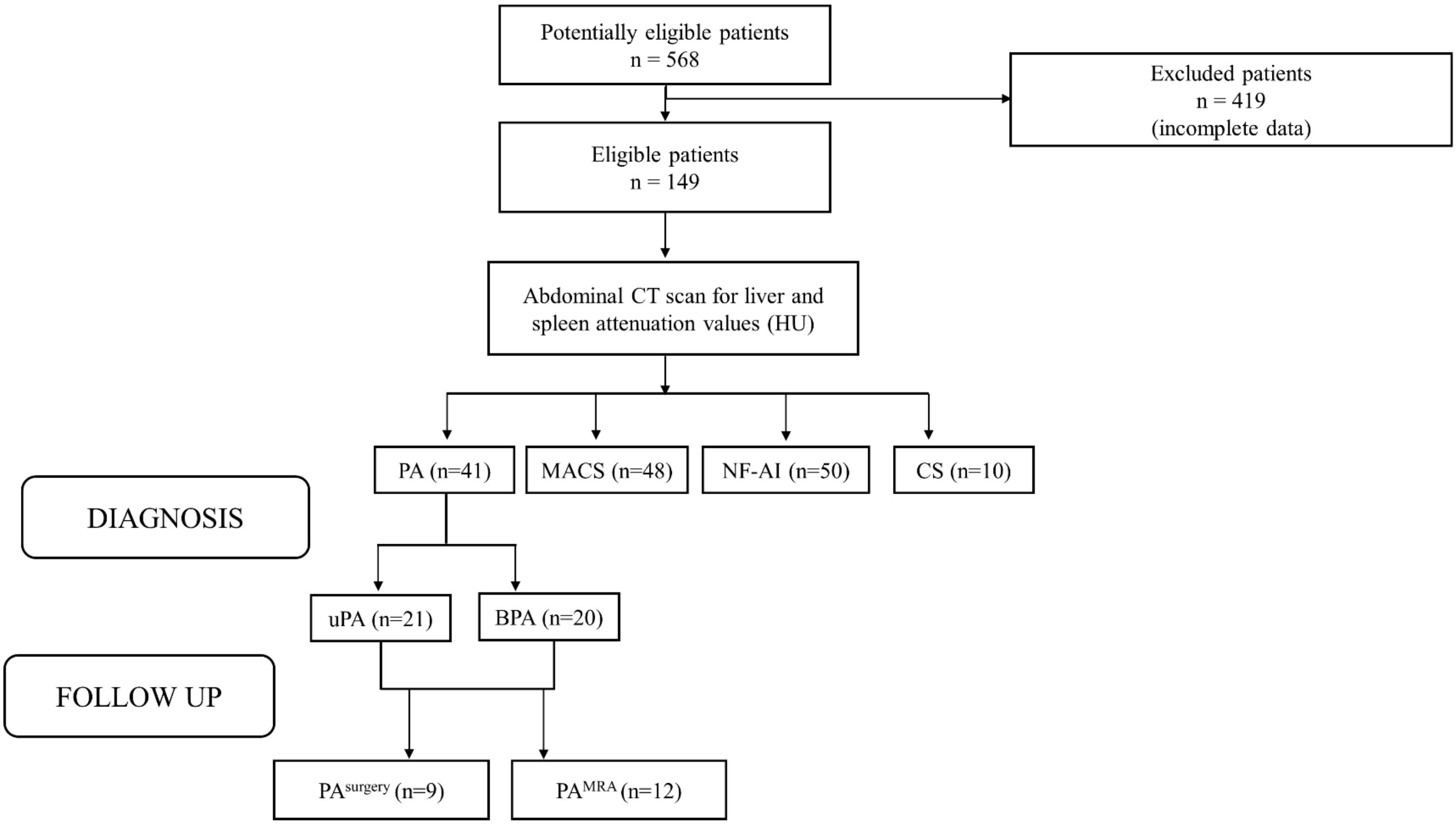

Supplementary Table S1. MAFLD distribution in the study population.

n patient	Adrenal disease	MAFLD					
		liver steatosis	glucose metabolism	dyslipidemia	hypertension	weight	MAFLD (yes/no)
1	PA	yes	normal	no	yes	oW	yes
2	PA	yes	DM	no	yes	oW	yes
3	PA	yes	IFG	yes	yes	oB	yes
4	PA	yes	normal	no	yes	oW	yes
5	PA	yes	normal	no	yes	oW	yes
6	PA	yes	normal	no	yes	oW	yes
7	PA	no	normal	yes	yes	normal	no
8	PA	no	IFG	no	yes	normal	no
9	PA	no	normal	yes	yes	oB	no
10	PA	yes	normal	no	yes	normal	no
11	PA	yes	/	yes	yes	oW	yes
12	PA	no	normal	no	yes	normal	no
13	PA	yes	IFG	yes	yes	normal	yes
14	PA	yes	normal	yes	yes	/	yes
15	PA	yes	normal	no	yes	oB	yes
16	PA	yes	normal	no	yes	oW	yes
17	PA	yes	normal	yes	yes	/	yes
18	PA	yes	normal	yes	yes	/	yes
19	PA	no	normal	no	yes	normal	no
20	PA	yes	normal	no	yes	oW	yes
21	PA	no	IFG	no	yes	/	no
22	PA	no	DM	no	yes	oW	no
dx	PA	no	IFG	no	yes	/	no
24	PA	no	IFG	no	yes	oW	no
25	PA	no	IFG	no	yes	normal	no
26	PA	yes	DM	yes	yes	oW	yes
27	PA	no	DM	no	yes	normal	no
28	PA	no	normal	no	yes	oW	no
29	PA	yes	DM	yes	yes	oB	yes
30	PA	yes	DM	yes	yes	/	yes
31	PA	no	normal	no	yes	normal	no
32	PA	yes	normal	yes	yes	oW	yes
33	PA	no	normal	no	yes	oW	no
34	PA	yes	DM	no	yes	/	yes
35	PA	no	normal	no	yes	normal	no
36	PA	no	normal	no	yes	/	no
37	PA	yes	normal	no	yes	normal	no
38	PA	no	normal	no	yes	/	no
39	PA	no	normal	no	yes	normal	no

n patient	Adrenal disease	MAFLD					
		liver steatosis	glucose metabolism	dyslipidemia	hypertension	weight	MAFLD (yes/no)
40	PA	yes	normal	no	yes	normal	no
41	PA	yes	IFG	yes	yes	/	yes
42	NF-AI	no	normal	no	yes	normal	no
43	NF-AI	yes	normal	yes	yes	oW	yes
44	NF-AI	yes	normal	no	yes	oW	yes
45	NF-AI	no	IFG	no	yes	oW	no
46	NF-AI	no	normal	no	yes	normal	no
47	NF-AI	no	normal	no	yes	normal	no
48	NF-AI	no	normal	yes	yes	oW	no
49	NF-AI	no	normal	no	yes	oW	no
50	NF-AI	no	normal	no	yes	normal	no
51	NF-AI	yes	DM	yes	yes	oW	yes
52	NF-AI	no	normal	no	yes	oW	no
53	NF-AI	no	normal	no	yes	normal	no
54	NF-AI	no	IFG	no	yes	oW	no
55	NF-AI	no	DM	no	yes	oW	no
56	NF-AI	no	normal	yes	yes	oW	no
57	NF-AI	no	normal	no	yes	oW	no
58	NF-AI	no	DM	no	yes	oW	no
59	NF-AI	no	normal	no	yes	oW	no
60	NF-AI	yes	IFG	yes	yes	oW	yes
61	NF-AI	no	normal	no	yes	normal	no
62	NF-AI	no	normal	no	yes	normal	no
63	NF-AI	no	DM	no	yes	oW	no
64	NF-AI	yes	normal	no	yes	oW	yes
65	NF-AI	no	normal	no	yes	oW	no
66	NF-AI	no	DM	no	yes	normal	no
67	NF-AI	no	normal	yes	yes	oW	no
68	NF-AI	no	normal	no	yes	oW	no
69	NF-AI	no	normal	no	yes	normal	no
70	NF-AI	no	DM	no	yes	normal	no
71	NF-AI	no	DM	no	yes	oW	no
72	NF-AI	no	IFG	yes	yes	normal	no
73	NF-AI	no	normal	no	yes	normal	no
74	NF-AI	no	normal	no	yes	oW	no
75	NF-AI	no	normal	no	yes	normal	no
76	NF-AI	no	normal	yes	yes	normal	no
77	NF-AI	no	normal	no	yes	normal	no
78	NF-AI	no	normal	no	yes	oW	no
79	NF-AI	yes	DM	yes	yes	oW	yes
80	NF-AI	no	normal	no	yes	normal	no

n patient	Adrenal disease	MAFLD					
		liver steatosis	glucose metabolism	dyslipidemia	hypertension	weight	MAFLD (yes/no)
81	NF-AI	no	normal	yes	yes	normal	no
82	NF-AI	no	normal	no	yes	oW	no
83	NF-AI	no	DM	yes	yes	normal	no
84	NF-AI	yes	IFG	yes	yes	oW	yes
85	NF-AI	no	normal	no	yes	normal	no
86	NF-AI	no	normal	no	yes	normal	no
87	NF-AI	no	normal	yes	yes	normal	no
88	NF-AI	no	normal	no	yes	normal	no
89	NF-AI	no	normal	yes	yes	normal	no
90	NF-AI	no	normal	no	yes	normal	no
91	NF-AI	no	normal	yes	yes	normal	no
92	MACS	no	DM	no	yes	normal	no
93	MACS	yes	DM	yes	yes	oW	yes
94	MACS	yes	normal	yes	yes	normal	yes
95	MACS	no	normal	no	yes	oW	no
96	MACS	no	normal	no	yes	oW	no
97	MACS	no	normal	no	yes	oW	no
98	MACS	no	IFG	no	yes	oW	no
99	MACS	no	normal	yes	yes	oW	no
100	MACS	no	DM	no	yes	oW	no
101	MACS	yes	DM	yes	yes	oW	yes
102	MACS	yes	DM	yes	yes	oW	yes
103	MACS	no	normal	no	yes	normal	no
104	MACS	no	normal	no	yes	normal	no
105	MACS	yes	normal	yes	yes	oW	yes
106	MACS	no	DM	no	yes	oW	no
107	MACS	no	normal	no	yes	normal	no
108	MACS	no	normal	no	yes	oW	no
109	MACS	yes	normal	yes	yes	oW	yes
110	MACS	no	normal	no	yes	normal	no
111	MACS	no	normal	no	yes	normal	no
112	MACS	yes	DM	yes	yes	normal	yes
113	MACS	no	DM	no	yes	oW	no
114	MACS	no	normal	no	yes	normal	no
115	MACS	no	normal	no	yes	normal	no
116	MACS	no	DM	no	yes	oW	no
117	MACS	no	DM	no	yes	normal	no
118	MACS	yes	DM	yes	yes	normal	yes
119	MACS	no	normal	no	yes	normal	no
120	MACS	yes	DM	yes	yes	normal	yes
121	MACS	no	normal	no	yes	normal	no

n patient	Adrenal disease	MAFLD					
		liver steatosis	glucose metabolism	dyslipidemia	hypertension	weight	MAFLD (yes/no)
122	MACS	no	normal	no	yes	normal	no
123	MACS	no	normal	no	yes	oW	no
124	MACS	no	normal	no	yes	oW	no
125	MACS	no	DM	yes	yes	normal	no
126	MACS	yes	normal	no	yes	oW	yes
127	MACS	no	normal	no	yes	normal	no
128	MACS	no	normal	no	yes	normal	no
129	MACS	no	normal	yes	yes	oW	no
130	MACS	yes	normal	no	yes	normal	no
131	MACS	no	DM	no	yes	oW	no
132	MACS	no	normal	no	yes	normal	no
133	MACS	no	normal	yes	yes	oW	no
134	MACS	no	normal	yes	yes	oW	no
135	MACS	no	normal	no	yes	normal	no
136	MACS	no	normal	no	yes	oW	no
137	MACS	no	normal	no	yes	normal	no
138	MACS	yes	DM	yes	yes	normal	yes
139	MACS	yes	normal	no	yes	oW	yes
140	CS	yes	IFG	no	yes	normal	yes
141	CS	yes	IFG	no	yes	oB	yes
142	CS	yes	IFG	yes	yes	oW	yes
143	CS	no	normal	no	yes	oB	no
144	CS	no	IFG	no	yes	oW	no
145	CS	no	normal	no	yes	oW	no
146	CS	no	normal	no	yes	oW	no
147	CS	no	IFG	yes	yes	oW	no
148	CS	yes	normal	no	yes	normal	no
149	CS	yes	IFG	yes	yes	oW	yes

Abbreviations: PA: primary aldosteronism, NF-AI: non functioning adrenal incidentalomas, MACS: mild autonomous cortisol secretion, CS: cushing syndrome, IFG: impaired fasting glucose, DM: diabetes mellitus, oW: overweight, oB: obesity.
